# GS-SMD server for steered molecular dynamics of peptide substrates in the active site of the γ-secretase complex

**DOI:** 10.1093/nar/gkad409

**Published:** 2023-05-19

**Authors:** Urszula Orzeł, Paweł Pasznik, Przemysław Miszta, Marcin Lorkowski, Szymon Niewieczerzał, Jakub Jakowiecki, Sławomir Filipek

**Affiliations:** Faculty of Chemistry, Biological and Chemical Research Centre, University of Warsaw, Warsaw, Poland; Faculty of Chemistry, Biological and Chemical Research Centre, University of Warsaw, Warsaw, Poland; Faculty of Chemistry, Biological and Chemical Research Centre, University of Warsaw, Warsaw, Poland; Faculty of Chemistry, Biological and Chemical Research Centre, University of Warsaw, Warsaw, Poland; Faculty of Chemistry, Biological and Chemical Research Centre, University of Warsaw, Warsaw, Poland; Faculty of Chemistry, Biological and Chemical Research Centre, University of Warsaw, Warsaw, Poland; Faculty of Chemistry, Biological and Chemical Research Centre, University of Warsaw, Warsaw, Poland

## Abstract

Despite recent advances in research, the mechanism of Alzheimer's disease is not fully understood yet. Understanding the process of cleavage and then trimming of peptide substrates, can help selectively block γ-secretase (GS) to stop overproduction of the amyloidogenic products. Our GS-SMD server (https://gs-smd.biomodellab.eu/) allows cleaving and unfolding of all currently known GS substrates (more than 170 peptide substrates). The substrate structure is obtained by threading of the substrate sequence into the known structure of GS complex. The simulations are performed in an implicit water-membrane environment so they are performed rather quickly, 2–6 h per job, depending on the mode of calculations (part of GS complex or the whole structure). It is also possible to introduce mutations to the substrate and GS and pull any part of the substrate in any direction using the steered molecular dynamics (SMD) simulations with constant velocity. The obtained trajectories are visualized and analyzed in the interactive way. One can also compare multiple simulations using the interaction frequency analysis. GS-SMD server can be useful for revealing mechanisms of substrate unfolding and role of mutations in this process.

## Graphical Abstract

**Graphical Abstract:**
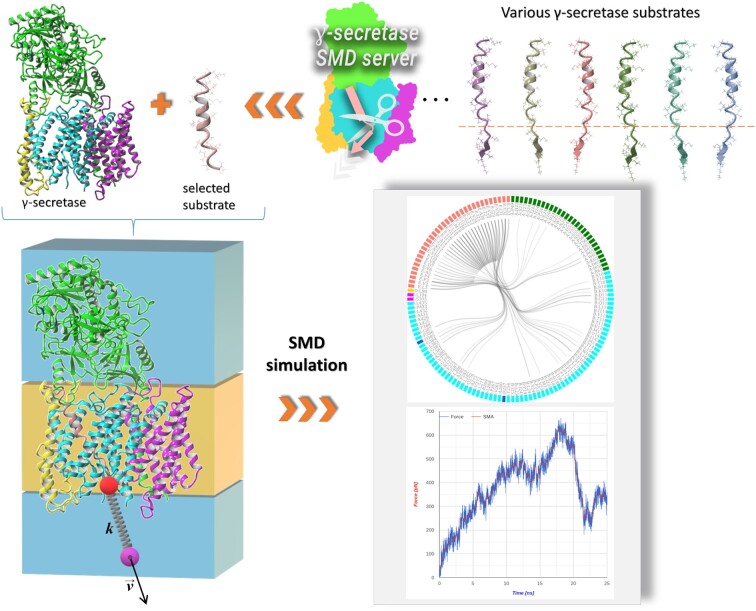

## INTRODUCTION

The γ-secretase (GS), which is a membrane protease complex, is well known for its role in Alzheimer's disease (AD), as it produces the pathogenic amyloid β (Aβ) peptide. AD is the leading cause of dementia and is characterized by a presence of amyloid plaques, composed mostly of Aβ peptides, in the brains of AD patients. The extracellular presence of Aβ plaques as well as soluble oligomeric forms of Aβ are considered the major cause of AD (1,2). However, it has been also demonstrated that GS plays a role in embryonic development (via Notch signaling (3)), adult tissue homeostasis, signal transduction and protein degradation (4). Among these substrates, the amyloid precursor protein (APP) has been the most studied since its cleavage and then trimming by GS leads to generation of aggregation-prone Aβ peptides. To explore the potential of GS as a therapeutic target, with the aim to develop inhibitors or modulators of GS activity, the studies on substrate-based peptides and peptidomimetics are essential (5). The currently used therapeutics against AD can temporarily ameliorate cognitive decline but are unable to stop or reverse the progression of this disease. Unfortunately, several drug candidates designed to block GS failed in clinical trials (6,7), therefore, further investigations involving GS substrates are required.

**Figure 1.**
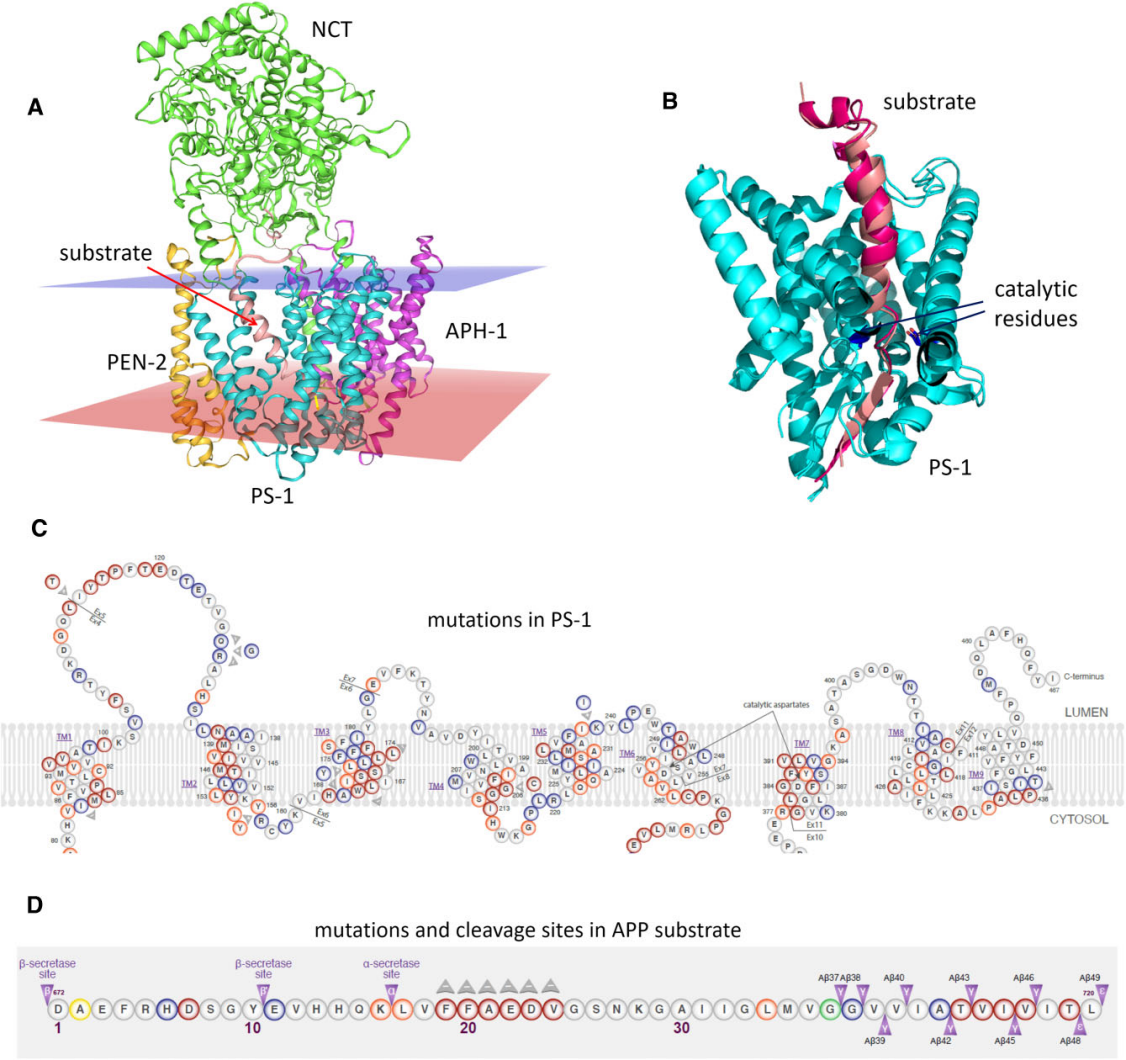
The γ-secretase (GS) complex and Alzheimer's disease (AD) mutations. (**A**) The cryo-EM structure (PDB id: 6IYC) (9) of GS: PS-1 in cyan, APH-1 in purple, NCT in green, PEN-2 in yellow, and the peptide substrate in salmon. The extracellular and cytoplasmic sides of the lipid bilayer are visualized by blue and red transparent rectangles, respectively. (**B**) Superimposition of cryo-EM structures of GS with APP (in salmon, PDB id: 6IYC) (9) and Notch receptor (in dark pink, PDB id: 6IDF) (10). Only PS-1 with substrate is shown. Some PS-1 helices are clipped to better show the substrate. The catalytic residues of PS-1 are shown in dark blue. (**C**) The AD mutations in PS-1. N-terminus and the long loop between transmembrane helices TM6 and TM7 are not shown. (**D**) The AD mutations and cleavage sites in fragment of APP substrate. The purple arrows show cleavage sites by secretases. The pathogenic mutations in dark red, likely pathogenic in red, benign in green, protective in yellow, and those with uncertain significance in blue. Mutations are based on ALZFORUM (Alzheimer Disease & Frontotemporal Dementia Mutation Database) [https://www.alzforum.org/mutations]. The material is copyrighted by AlzForum Foundation Inc.

GS complex consists of four membrane proteins: the catalytic subunit presenilin (PS-1), PEN-2, APH-1 and nicastrin (NCT) (Figure 1A) (8). The recent cryo-EM structure of GS with the APP substrate was determined with a very good resolution, 2.6 Å (PDB id: 6IYC) (9), allowing to see atomic details of the structure. Published in the same year the structure of GS with another substrate, being a fragment of Notch receptor (PDB id: 6IDF) (10), revealed not only the same secondary structure of GS but also the same helical secondary structure of the substrate, located in the same binding site of GS (Figure 1B). The mechanism of gradual movement of the trimmed substrate down to the active site of GS was also proposed (10). Based on this mechanism we employ a threading of the substrate sequence into 6IYC structure of GS complex to mimic the structure just after the cleavage, to study unfolding of the substrate for the next cut, which is done by the catalytic aspartate residues D257 and D385 of PS-1. Although the structure of GS has been solved, it is still not known how GS recognizes its substrates and how the substrates are being unfolded in the active site.

Due to its complexity and importance, the mechanism of substrate processing by GS was a subject of many investigations, e.g. recent papers (11–15), including theoretical calculations. The studies also involve the effect of mutations in GS, mostly in PS-1 being the catalytic core of GS complex, since it contains most of the known AD mutations (16,17). Nearly 300 causative mutations represent ∼90% of all mutations associated with an aggressive AD form (Figure 1C). PS-1 plays an essential role in neural progenitor maintenance, neurogenesis, neurite outgrowth, synaptic function, neuronal function, myelination, and plasticity. Therefore, mutations in PS-1 might cause aberrant neuronal signaling and synaptic dysfunction, contributing to neurodegeneration during the initial stages of AD (18). These dysfunctions can also be caused by mutations in the GS substrates, and especially in APP (Figure 1D).

The set of GS substrates includes Notch-1 to Notch-4 receptors, E-cadherin, alcadeins, CD44 receptor, GHR (growth hormone receptor), and VEGFR-1 (receptor protein-tyrosine kinase). The cleavage sites by GS are determined for only 23 substrates, however, in GS-SMD server, we propose a position for the initial cleavage for all substrates based on the multiple sequence alignment. Furthermore, since the substrate sequence can be modified, or even entirely new sequence can be entered manually, any substrate is possible. The multiple cleavage sites are well known for APP substrate (arrows in Figure 1D) leading to a large set of various Aβ peptides – after initial cleavage APP is trimmed every three/four residues, leading to Aβ_40_ and Aβ_42_, which are the major components of amyloid plaques (1,2,19). The sizes of amino acid side chains in the trimmed segments of APP, have been recently reported to contribute to APP binding and cleavage by GS (20), so possibly, the similar effect of trimming can be observed for other, less studied, substrates.

**Figure 2.**
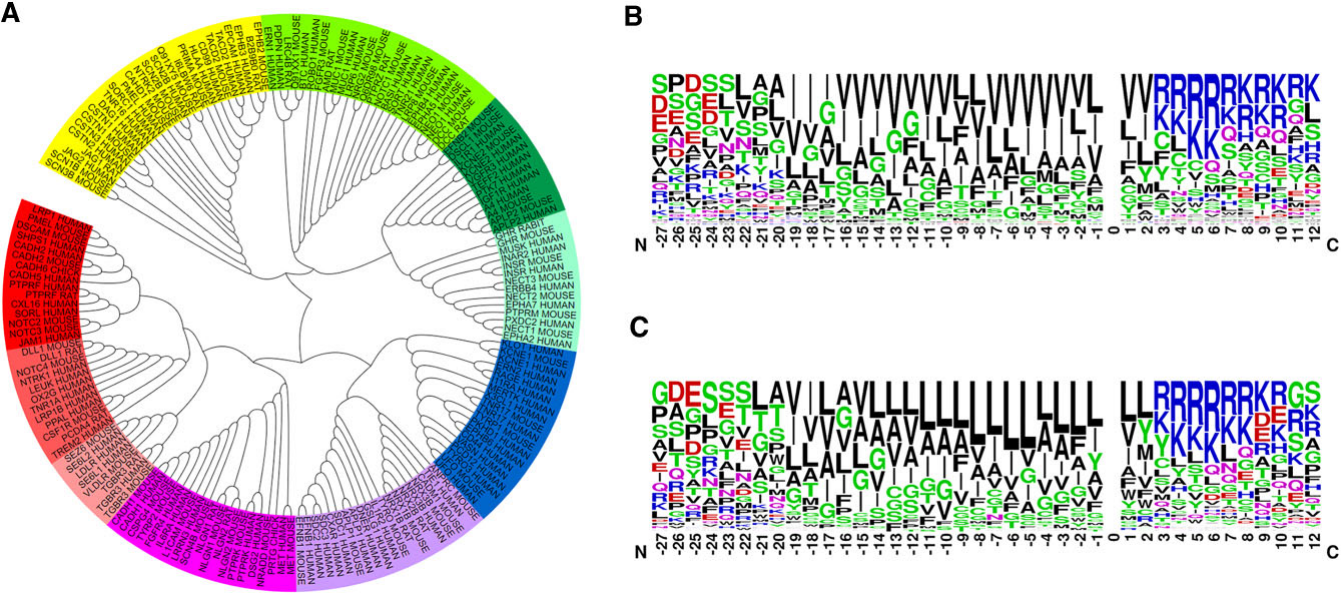
Comparison of transmembrane (TM) sequences of all known substrates of GS. (**A**) Dendrogram showing similarities and differences in TM sequences of GS substrates. The division into two subgroups is clearly visible. The substrates are marked by their UniProtKB entries. (**B**) The sequence logo for the substrates from the upper part of dendrogram. (**C**) The sequence logo for the substrates from the lower part of dendrogram. The sequences of substrates, each of 39 amino acid length, are aligned based on the cleavage site, which is No. 0 on the X axis (if the cleavage site is not determined yet, the alignment is based on the hydrophobicity of TM region). The transmembrane regions can differ among the substrates but approximately they are located between residues nos. –19 and 2.

In total, GS cleaves over 170 single-pass membrane proteins of type-I (with its N-terminus on the extracellular side of the membrane). Comparing the transmembrane (TM) domain sequences of the substrates one can find interesting dependencies. Dendrogram of their TM sequences is divided into two parts (Figure 2A). Analyzing them separately one can find that the substrates from upper part of dendrogram mostly have valine residues in their TM domain (Figure 2B), while the substrates from lower part of dendrogram predominantly have lysine residues (Figure 2C). Such comparisons indicate that the interactions between the substrates and GS are different for different substrates, TM domain is not only an anchor, and one can effectively study its properties and interactions by unfolding the substrate in the active site of GS. The interactive dendrogram in GS-SMD server allows selecting the substrate and includes links to the protein sequence UniProt database (21) and the whole length protein structure AlphaFold database (22,23).

Spontaneous unfolding of GS substrates in the active site takes a very long time since the enzymatic activity of GS is extremely low. Therefore, in our server we employ the steered molecular dynamics (SMD) simulations to unfold the cleaved C-terminal part of the substrate. The method of protein unfolding, known as a single molecule force spectroscopy, is widely used (24–26) to reveal the internal forces responsible for protein stability and for studying dynamics of protein folding. Here, we employ the process of unfolding to mimic the natural event of substrate unfolding in the active site of GS during preparation for the next cut. In GS-SMD server it is also possible to combine unfolding events using different pulling directions, velocities and spring constants in subsequent SMD simulations, by taking the structure of the GS-substrate complex derived from the previous simulation to the next one. One can also run MD simulation without pulling on the server to relax the extended/unfolded structure of the substrate. Knowledge of unfolding events and interactions that can be obtained by our server can facilitate development of substrate-specific inhibitors or modulators.

The GS-SMD server can be useful for a broad range of scientific workers including molecular modelers and experimentalists, as well as students. The server is friendly to be used by inexperienced people, and the extensive tutorial explains the procedures and analyses in detail. According to our knowledge there are no similar web servers for conducting SMD simulations for proteins or web servers for molecular dynamics of GS.

## MATERIALS AND METHODS

### Web interface

The web interface is built in Bootstrap 4.4 while the visualization of structures is performed by NGL Viewer (27,28). Circular plots of molecular interactions are created in flareplot (trajectory data is processed by time-flare script) and the interactive linear plots are implemented in Google Charts. Visualization of a pulling direction by an arrow is implemented in the SMD form on the MD&mut input page and also on Analysis page. The progress bar showing a status of the job and the remaining simulation time is displayed after starting the SMD/MD simulation.

### Backend of the server

The GS-SMD server is processing jobs using the Celery message passing queue. Web interface, database queries and server logic are implemented with the Django framework. The SMD simulations are performed in an implicit water and membrane environment using the IMM1 (29) method implemented by us in NAMD (30) v.2.14 running on CUDA-enabled GPUs server.

### The implicit environments

The SMD/MD simulations performed at GS-SMD server are employing the implicit heterogeneous environment which includes both solvent and membrane media. The approach is based on the implicit membrane methodology IMM1 (29) being the extension of EEF1 method (31). IMM1 is parameterized for CHARMM19 force field combined with a Gaussian model for the solvation free energy. CHARMM19 is the united-atom force field without aliphatic hydrogen atoms which additionally diminishes the number of atoms in the system. The solvation model calculates how the neighboring atoms affect the solvation energy of a given atom by excluding solvent from the surrounding space. The membrane is implemented as a slab, parallel to the *xy* plane centered at *z* = 0, with a smooth transition of solvation parameters for each atom at the interface using the continuous switching function. The transition region between the hydrophobic core and the water environment is about 0.6 nm wide, which is in agreement with experimental data of lipid bilayers. All the necessary details of the underlying methodology of implicit environments are described in our earlier paper on GPCRsignal server (32) which was constructed for studying dynamical interactions between GPCRs (G protein-coupled receptors) and their effector proteins (G proteins and arrestins). In SMD/MD simulations, in the implicit water-membrane environments, there are no explicit water molecules and no lipids. It makes simulations faster but there are some drawbacks, for instance, there is no possibility of bridging hydrogen bonds by water molecules, however, a formation of direct hydrogen bonds is feasible.

### Curation of GS complex and threading

For SMD/MD simulations we have employed cryo-EM structure of GS with APP substrate (PDB id: 6IYC) (9). The N-terminus of the substrate is not visible except for residues 1–6 interacting with NCT. The missing unfolded five residues 7–11 were reconstructed. The side chains that were only partially visible in the GS complex were also reconstructed. The hydrogen atoms were added to the whole complex at pH 7 and the hydrogen bond network was optimized. The mutation of one of the catalytic residues, D385A, introduced to prevent substrate cleavage by GS, was reversed. Then, the restored residue D385 was protonated to create the proper catalytic environment. Only one catalytic residue was protonated to comply with the cleavage mechanism of the aspartyl protease. The lacking in cryo-EM structure N-terminal part of PS-1 (residues 1–72) as well as the long loop between TM6 and TM7 helices (residues 292–375) of PS-1 were not reconstructed as they are not necessary for studying unfolding events in the active site of GS. The GS-APP structure without a loop between TM6 and TM7 helices of PS-1 was recently used for GaMD (Gaussian accelerated molecular dynamics) simulations. That structure proved to be stable during the simulation of 2000 ns (11).

The currently available experimental structures of GS (PDB id: 6IYC, 6IDF, 6LQG, 6LR4, 7C9I, 7D8X) are nearly identical including the same positions of side chains of GS subunits. Therefore, we decided to use only one structure of GS in our server. If new, and different, experimental structures of GS will be available, they will be included in the server as input structures. We do not allow to use input structures generated by users, as there is no verification if the input structure is really GS, and also the threading procedure for such structure would be problematic. In order to study unfolding of APP after the cleavage, we cut out the residues behind the cleavage site, receiving the substrate consistent with the Aβ_49_ fragment, however, its N-terminus is shortened, because 33 residues of the substrate, and not 49, are visible in the cryo-EM structure 6IYC. As it was found by Bhattarai *et al.* (11), AICD (APP intracellular domain) is necessary for initial pulling the C-terminus of the substrate to set it up for the next cleavage step—in our server this is achieved by direct pulling of C-terminus of the substrate. To study other Aβ fragments of APP and also other GS substrates we employ the threading procedure with the sequence of a given substrate applied to the structure of GS-APP (PDB id:6IYC). Threading is conducted in open-source PyMOL by mutating all residues of initial APP substrate to the selected substrate/mutant—the sequences are aligned to impose their cleavage sites at C-terminal ends. During mutagenesis PyMOL selects rotamers to maximally avoid steric clashes. Other short contacts are removed during the optimization procedure: 3 × (1000 steps of conjugated gradient minimization of energy and 2 ps MD simulation).

### SMD and MD simulations

**Figure 3.**
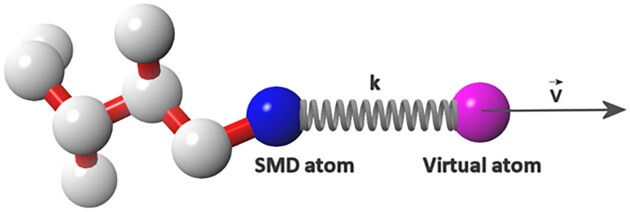
The scheme of SMD simulations with constant velocity. In the GS-SMD server the Cα atom of the C-terminal residue of the substrate is defined as SMD atom. This atom is pulled by a virtual atom attached to the SMD atom by a virtual spring with a spring constant k.

For SMD/MD simulations in the implicit environments the Langevin dynamics is used with a damping constant of 40 ps^−1^ and with 2 fs time step whereas all bond lengths are constrained using SHAKE (33) algorithm. The simulations run at temperature 298 K with nonbonded interactions cutoff at 14 Å and switching at 12 Å. Originally, the IMM1 method was implemented in CHARMM program (34), however, we ported this method to NAMD (30) to take advantage of GPGPU and parallelization. To unfold the substrate in the binding site of GS a force is applied to C-terminal part of the substrate (so called SMD atom) by a virtual spring, and its magnitude depends on the virtual spring force constant (*k*) and the velocity of the virtual atom (*v*), while the force vector is always parallel to the pulling vector of the virtual atom. The force vector coordinates, as well as coordinates of SMD atom are monitored and recorded throughout the simulation. The resistance experienced by the molecule is expressed as a force required to overcome it (user specifies the pulling velocity and the spring constant), and it is also possible to calculate the work/energy associated with each unfolding event. In case of selecting several residues for pulling the SMD atom represents center of mass of Cα atoms of selected residues. A virtual atom moving with a constant velocity is connected to the real atom (SMD atom – in our simulations it is by default Cα atom of C-terminus, but it can also be the center of mass of selected residues) of the studied molecule by a virtual spring (Figure 3).

To compare SMD simulations in implicit and explicit systems we conducted SMD simulations of the GS-APP complex (shown as an example system in GS-SMD—it corresponds to the Aβ_49_ fragment of APP) in full membrane and water (temperature 298 K, pressure 1 bar). For explicit solvent simulations, the GS-APP complex was embedded in the membrane using CHARMM-GUI (35,36). The membrane composition used reflects the membranes of brain tissues affected by AD (37) including glycerophospholipids (POPC), cholesterol, sphingolipids (DSM) and diacylglycerol (DAGL) in a ratio 35:40:15:1. To the protein-membrane system the solvent was added: water type TIP3P and neutralizing sodium and chloride ions at concentration 0.15 M. The 25 ns long SMD simulations were conducted in NAMD (30) program with four repetitions. The pulling speed *v* = 0.1 m/s was applied to the Cα atom of C-terminus of the substrate and the spring constant *k* = 1.0 kcal/mol/Å^2^ was employed both for implicit and explicit solvent simulations. The default pulling direction was used – it is based on the expected final position of the substrate C-terminus that forms a β-sheet with PS-1 as it is observed in 6IYC cryo-EM structure.

### Workflow

- Selection of the simulated system (the whole GS complex / the membrane part of GS / PS-1).
- Selection of substrate and the substrate sequence, the position of cleavage, and introduction of optional mutations to the substrate and GS.
- Selection of parameters for SMD simulations including pulling direction, velocity and spring constant. Possibility to select several residues for pulling. Alternatively, to run MD simulation without substrate pulling to relax the extended/unfolded structure of the substrate.
- Threading procedure: a sequence of a selected substrate is applied to the structure of GS-APP complex.
- Running SMD/MD simulation of GS-substrate complex in the implicit environments; preparation of plotting data and downloadable archive after completion of simulation.
- Interactive visualizations: (i) the protein complex structure with pulling arrow, (ii) the interactions of substrate with GS on interactive flareplot, (iii) plots of force, work/energy, and the secondary structure of the substrate versus time.
- Possibility to use output structure (any frame) as an input for continuation of SMD/MD of the same GS-substrate pair with different SMD/MD parameters.
- Optionally, comparison with other simulations using the frequency difference analysis.

## RESULTS

### Description of input

The input data for SMD simulations of the GS-substrate complex is prepared on the MD&mut page. It begins with a selection of one of the three curated structures: (i) the whole GS complex, (ii) the membrane part of GS and (iii) PS-1. Then, the user selects the substrate to bind to GS from a list of substrates with defined cleavage sites (4). The main, or initial, cleavage site is marked with an asterisk. In the next line, showing the membranous sequence of the selected substrate, user can modify the cleavage site by moving the asterisk to another position and introduce optional mutations, deletions and insertions in the sequence. Only capital letters are allowed. The final sequence fragment that will be used for SMD simulation is highlighted below the input line. The fragment must be minimum 15 residues long and maximum 33 residues long because no more substrate residue is visible in the cryo-EM structure 6IYC (9) taking into account residues from the cleavage site to the N-terminus. Pressing the [Example input] button loads the whole GS complex with APP substrate and the initial cleavage site – that fragment is known as Aβ_49_, however, its N-terminus is truncated by 16 residues non-visible in the cryo-EM structure 6IYC. There is a possibility to introduce mutations into any subunit of GS complex by specifying mutations in each chain – available ranges of residues are specified. The [Default Mutations] button introduces three mutations in chain B (PS-1) which are the early onset AD mutations (18).

Parameters for SMD simulation, specified by user using sliders or input fields, include: pulling velocity (range from 0.01 to 0.5 m/s, default 0.1 m/s), spring constant (range from 0.1 to 10 kcal/mol/Å^2^, default 1.0 kcal/mol/Å^2^), and a pulling direction by changing the position (*x*, *y*, *z*) of the end of the arrow. The start of the arrow is located at the Cα atom of C-terminus of the substrate (or center of mass of Cα atoms of selected residues). The arrow is visible at the adjacent interactive window showing the structure of the GS-substrate complex. The four final parameters for SMD simulations are: the total length (range 5–25 ns, default 15 ns), number of frames (range 10–200, default 50), the membrane thickness (20–40 Å, default 31 Å, which is a value from OPM database (38) for GS complex), and number of tasks to run (1–4, default 1). Since SMD and MD simulations are stochastic processes it is better to conduct more simulations to obtain statistically valid results. After confirming the entered data, the user will see a progress bar of the currently running simulation (or simulations in case of parallel tasks).

The extent of substrate unfolding depends on many factors: length of simulation, pulling velocity, spring constant (a measure of the stiffness of the spring), direction of pulling, and obstacles on the way of pulling. For APP, the maximal length of simulation available on the server, 25 ns (default is 10 ns), is far enough to obtain a conformation for next cut. Such simulation is shown in menu as Example 1. User can select any residue (its Cα atom) for pulling in any direction, and it is also possible to select more than one residue for pulling. This could allow to simulate e.g. tilting of the substrate – such tilting can be seen in Example 2 when residues from N-terminus of the substrate are selected. In Example 3, user can do a comparison of four SMD trajectories and conduct the interaction frequency analysis to see similarities and differences in substrate interactions.

### Browsing and analyzing the single trajectory

**Figure 4.**
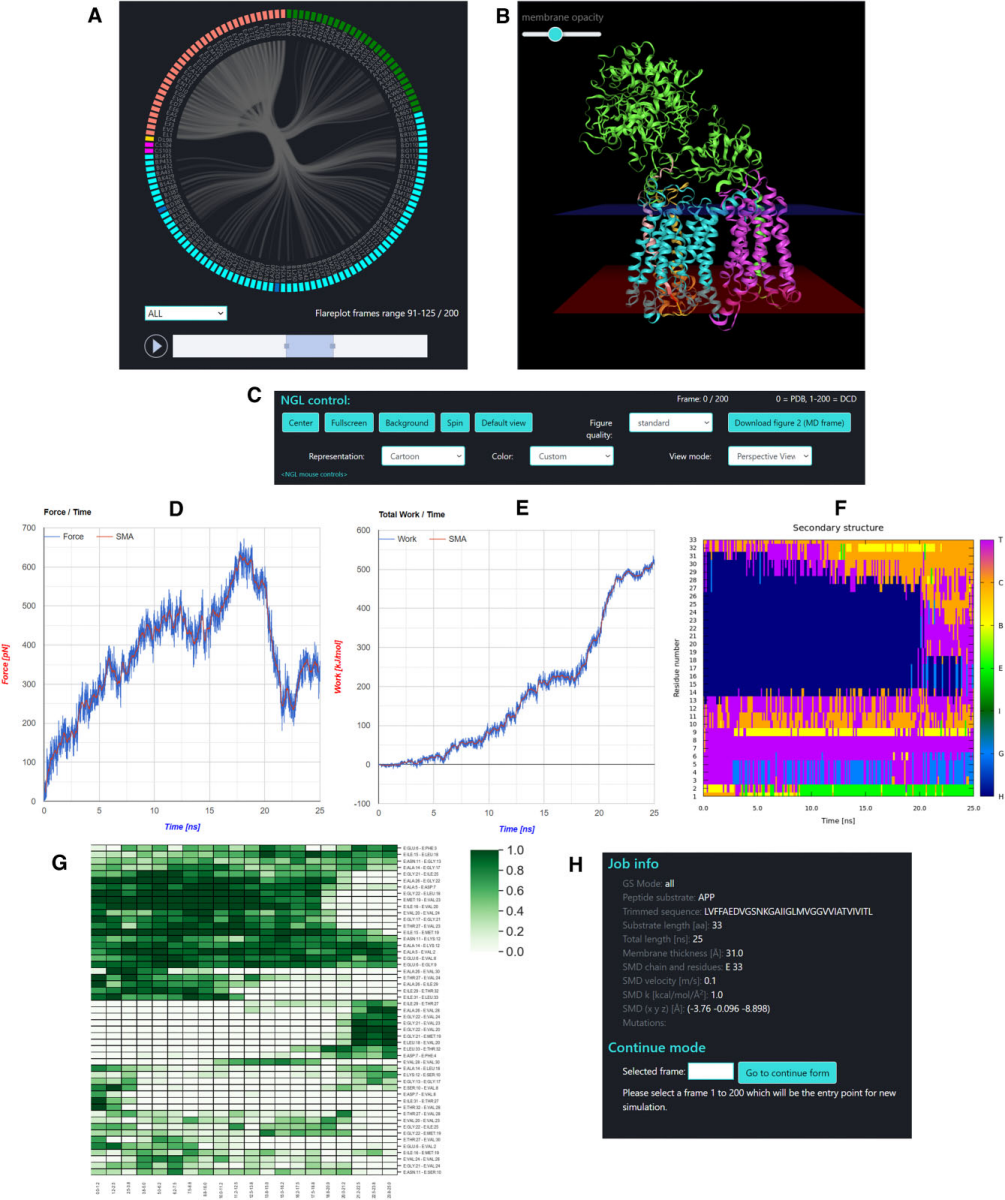
The interactive output windows for analysis of a single trajectory. (**A**) Flareplot displays various types of interactions of substrate (in salmon) with GS. The trajectory slider enables averaging over time. Colors in flareplot correspond to colors of GS subunits. PS-1 catalytic residues in the flareplot diagram are colored dark blue. (**B**) Structure visualization window. The pulling vector is shown as a yellow arrow and the spring pulling the C-terminus of the substrate as a red line. (**C**) The controls used for the visualization window. (**D**) Force versus time for conducted SMD simulation. SMA is a simple moving average. (**E**) Total work/energy versus time. (**F**) Changes in the secondary structure of a stretched substrate over time. Abbreviations: H – α-helix, G – 3_10_-helix, I – π-helix, E – strand, B – bridge, C – coil, T – turn. (**G**) The heatmap of internal hydrogen bonds of the substrate during its unfolding. (**H**) Job information and a form for the Continue mode.

After completing the SMD simulation, the user is redirected to the Analysis page. This page includes visualization of GS-substrate contacts via interactive flareplot (Figure 4A) which can display various types of interactions (all, salt bridges, hydrogen bonds, aromatic, van der Waals and hydrophobic). In flareplot, the substrate internal contacts are also visualized to show changes in its structure during unfolding. The adjacent window contains an NGL viewer for visualization of the obtained trajectory (Figure 4B). The visualization window control panel provides access to a selection of representations, coloring modes and viewing modes (Figure 4C). In the NGL viewer, the user can see the obtained trajectory with additional information: visualization of membrane with adjustable opacity and visualization of pulling by an arrow that represents the pulling vector. The arrow is connected by a spring to selected Cα atom (or center of mass of Cα atoms of residues selected for pulling). In the second part of the Analysis of single trajectory page, the graphs showing force (Figure 4D) and work/energy (Figure 4E) as a function of time, as well as changes in the secondary structure of the stretched substrate (Figure 4F), the heatmap of internal hydrogen bonds of the substrate during its unfolding (Figure 4G), and information about the task along with the form for the Continue mode (Figure 4H) are displayed. The user interested in continuing the simulation can do it by selecting the trajectory frame at the bottom of the Analysis page and pressing the [Go to continue form] button. The Continue page appears with similar selectable parameters as on the MD&mut page but without changing the GS–substrate pair and mutations (the structure from the selected frame must be preserved). In this way, one can explore different ways to unfold the substrate.

Optionally, the user can download the result files package. Package includes: trajectory file (dcd), structure files (pdb and psf), contacts list, contacts frequencies files and flareplot data. It also contains data files for force and work graphs and the starting position of the SMD atom. Job results are to be available on the server at least two weeks after completion of the job. The user can see the results and analyze them using the link (token) copied from the progress bar page, copied from the top of the Analysis page, sent by mail, or included in the job description file in the results package. It is also possible to share the obtained trajectory with other users. This can be done using the [Make this job available to public] button on the results page (available immediately after the simulation is finished or later with the simulation token). The published simulations are visible under the ‘Shared jobs’ menu item. They will not be removed after two weeks.

### Comparison and analysis of multiple trajectories

**Figure 5.**
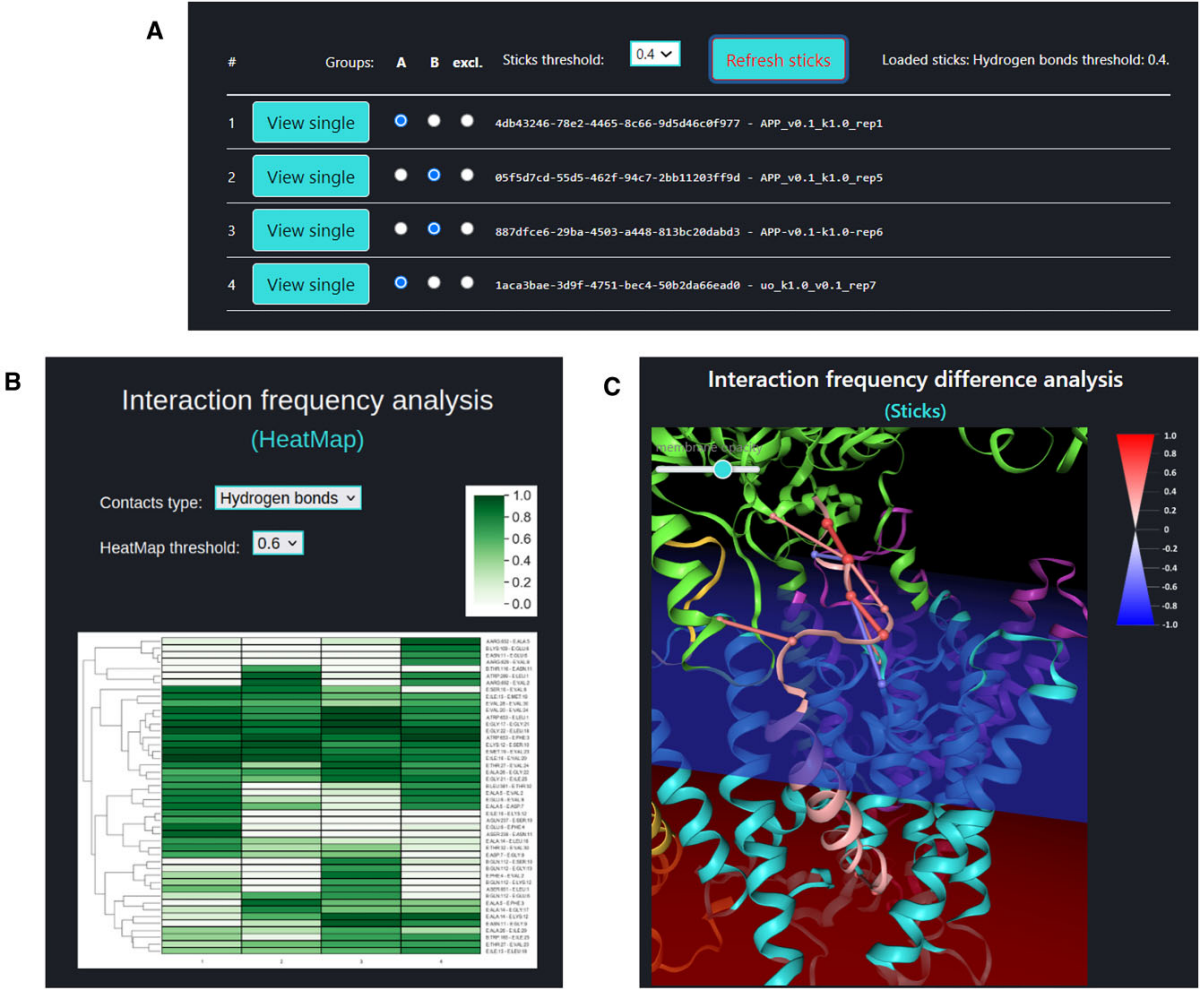
The analysis of multiple trajectories. (**A**) Grouping of trajectories into sets A and B for analysis of differences in residue–residue interactions involving the substrate. (**B**) The interaction frequency analysis for the particular interaction type (hydrogen bonds, salt bridges, aromatic) for all trajectories for a chosen threshold. (**C**) The residue-residue interaction frequency difference analysis for the trajectories selected in sets A and B for the chosen threshold (sticks threshold).

When several simulations are completed, one can compare them using ‘Compare jobs’ page to select required trajectories by their tokens. Several residue-residue interaction types can be analyzed (hydrogen bonds, salt bridges, aromatic) for studies of the substrate interactions (internal interactions as well as to GS subunits). The user can select multiple trajectories into groups A and B, to be compared with one another (Figure 5A). It is also possible to review each trajectory in a ‘Single Trajectory’ mode by clicking [View single] button next to trajectory number.

The HeatMap (Figure 5B) visualizes the frequencies of residue-residue interactions (contacts) involving the substrate (chain E) calculated for each of the trajectories included in the comparison. The contact frequency values span between 0 and 1. Frequency 1.0 means that a certain interaction is observed in 100% of the trajectory frames, frequency 0.5 is observed in 50% of the trajectory frames, frequency 0% means that this interaction was not found in particular trajectory but this interaction is collected because it exists in other compared trajectories and is equal or larger than the ‘HeatMap threshold’ value.

The Interaction frequency difference analysis (so called Sticks – Figure 5C), identifies only those interactions for which the frequency difference (between trajectory sets A and B) are equal or greater than the ‘Sticks threshold’. The stick color represents the value of a frequency difference for that interaction between trajectory sets A and B. Sticks threshold (its default value is 0.4) represents a minimal difference between frequencies of a certain interaction in sets A and B required to show this interaction as a stick and include the frequency difference of this interaction in a table. The lower the threshold the more interactions will be included.

Due to stochastic nature of conducted simulations several of them are required to obtain reliable results. The more trajectories each set contains, the more likely it is that a frequency difference of a certain interaction between those two sets is not random, but is rather caused by the change of simulation conditions: different direction of pulling, spring constant, membrane thickness, GS mode (whole system or part of it), mutation, etc. For comparison of mutated substrates, the interactions formed by mutated and unmutated residue will be interpreted as two distinct interactions, yielding two separate rows in heatmap and two separate sticks.

### Tutorial and timeline

The GS-SMD web server includes an extended tutorial that guides the user step by step on how to perform SMD simulations. The tutorial starts with a brief explanation of the SMD method, which is the main method of GS-SMD server. Description of SMD is accompanied by a schematic illustration of the method and the sample graphs (force and work/energy as a function of time) obtained from the server. Then, all menu pages and the features present in the GS-SMD server are described in detail, and each of them is illustrated with appropriate screenshots. The input parameters are explained and the job submission process is illustrated with exemplary input data. A table of all GS substrates with defined cleavage sites (4) is provided with links to the Uniprot database (21). In addition, all the tools for analyses and visualizations available in GS-SMD are presented with exemplary results. There is also a Timeline page with all available GS structures in apo form, with substrates, and inhibitors/modulators.

To facilitate interpretation of SMD simulations by inexperienced users, we have added new data in the tutorial showing the changes in the hydrogen bond network in APP. This was done as an example of single SMD trajectory analysis. By analyzing the SMD trajectory, the user can identify the hydrogen bonds that form/break during the simulation. As such analysis is highly dependent on the GS-substrate/mutant pair, it is not part of the server and should be performed individually.

## DISCUSSION

### Performance and comparison to all-atom SMD simulations in explicit environments

**Figure 6.**
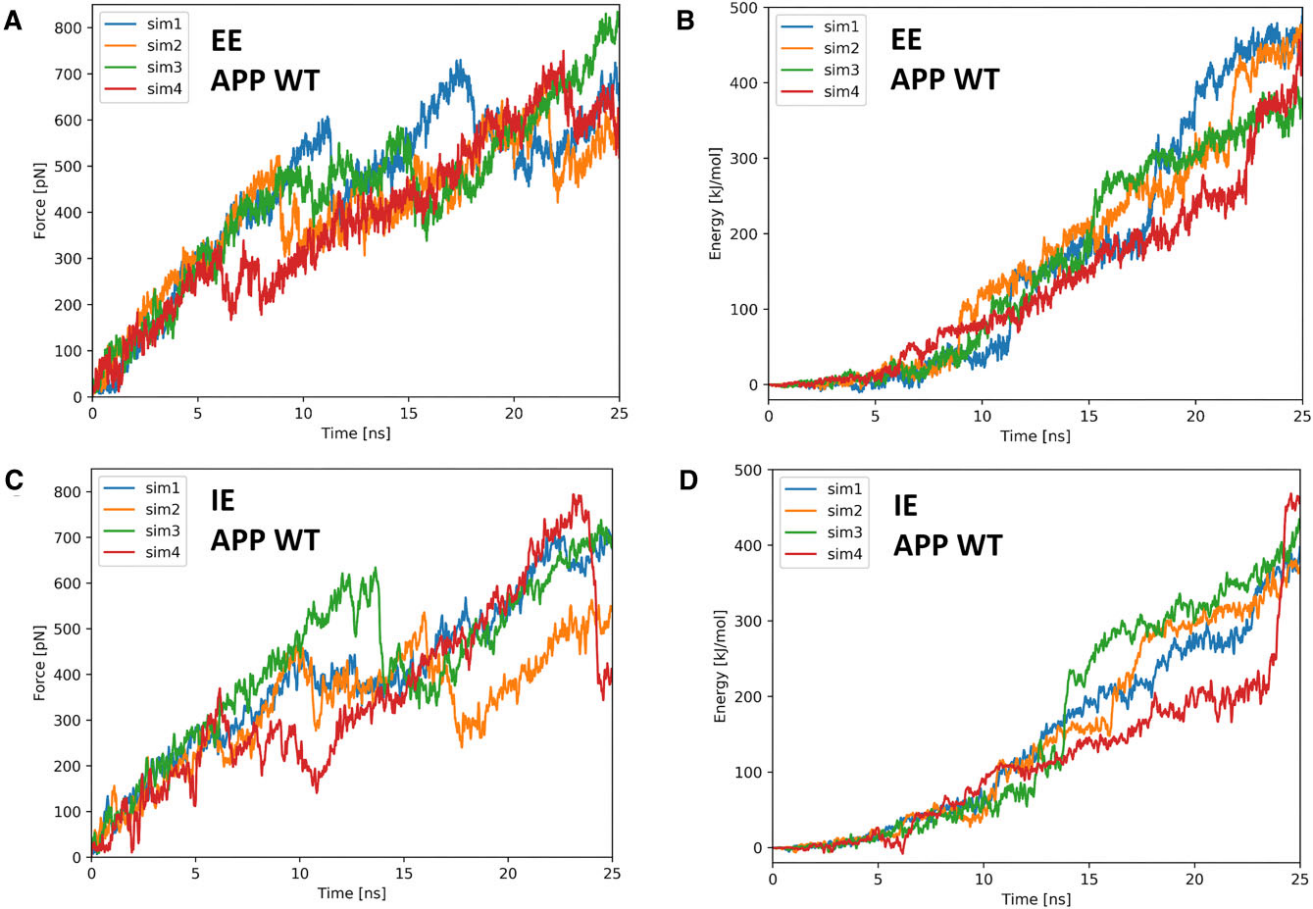
Comparison of SMD simulations conducted in explicit (EE) and implicit (IE) environments. All SMD simulations of the GS-APP WT complex were conducted with the same parameters: *v* = 0.1 m/s, *k* = 1 kcal/mol/Å^2^, and pulling in the default direction (definition of default pulling direction in Methods). (**A**) Force versus time in EE. (**B**) Work/energy vs. time in EE. (**C**) Force versus time in IE. (**D**) Work/energy vs. time in IE.

The GS-SMD server allows to perform the peptide substrate unfolding in the GS active site. Depending on the protease structure used (all GS complex, membrane part or only PS-1) the SMD simulation of 25 ns is completed in approximately 6, 4 and 2 h, respectively. The same simulation in explicit membrane and water is 5–6 times longer, not counting system setup time. Considering that multiple SMD simulations of the same system are needed to get a more complete unfolding picture, the reduction in time required is significant. Comparing the force and work/energy versus time graphs obtained in explicit (Figure 6A, B) and implicit (Figure 6C, D) environments for GS–APP complex, it can be seen that the graphs are similar in the same force and work/energy ranges. We also observe breaking of hydrogen bonds in the substrate helix and formation of 3_10_ helix at the C-terminus of the substrate in both implicit and explicit environments. This indicates that the results obtained on the server are reliable and can be used to investigate unfolding events.

### Comparison to experimental results

**Figure 7.**
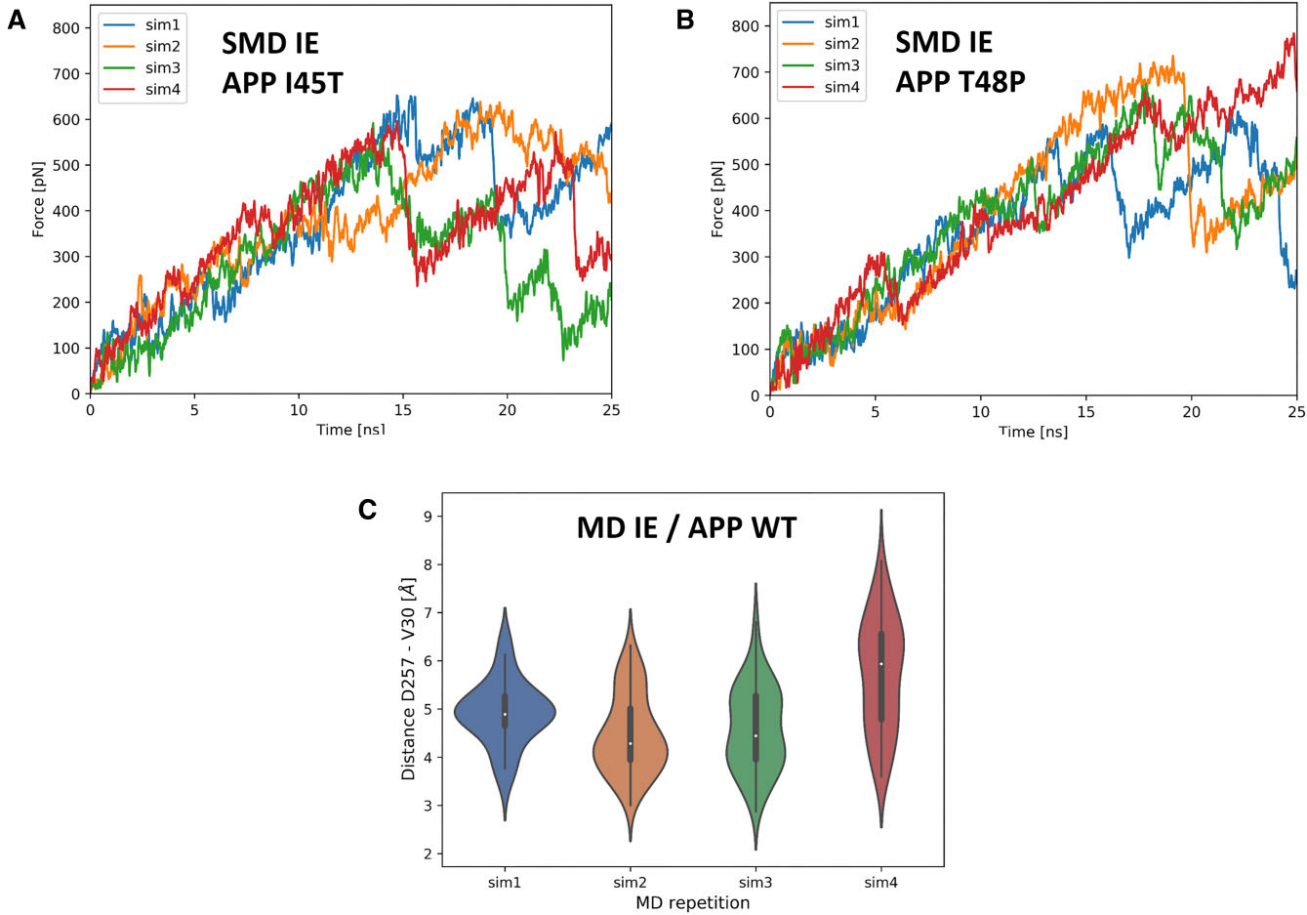
SMD and MD simulations conducted in implicit (IE) environments. (**A**) Force versus time for I45T APP mutant. (**B**) Force versus time for T48P APP mutant. APP mutants were selected for comparison with experimental data from (11). (**C**) The violin plots for a distance of the scissile bond (carbonyl group of V30 residue) to one of catalytic residues (D257) in 25 ns MD simulations of GS-APP WT complex in Continue mode for SMD simulations from Figure 6. The MD simulations were conducted once for each SMD simulation from Figure 6C (implicit environments).

To compare SMD simulations with the experimental data, we analyzed *F*/*t* plots from SMD simulations for wild type APP and its mutants, I45T and T48P—these mutants were studied by Bhattarai *et al.* (11), and found to hinder unfolding. The *F*/*t* plots (Figure 7AB) demonstrate that APP mutants require longer pull time to reach the first event of unfolding as indicated by the first significant decrease in force. This is especially true for the T48P mutant (T32P on GS-SMD server because of shorter substrate) (Figure 7B) compared to wild type APP (Figure 6C). As a result, the SMD method implemented on the server is sensitive enough to obtain reliable results consistent with the experimental data. However, one should remember that the simulations are stochastic processes and even under the same conditions, different trajectories could be obtained, therefore several SMD simulations are required in each case. Also, comparing different substrates can give erroneous results. Therefore, we recommend making comparisons only for the same substrates with various mutations in the substrate or/and in GS.

In GS-SMD server it is also possible to turn off SMD mode and run MD simulation without substrate pulling. In this case all SMD parameters are disabled. We verified for APP substrate, that after selecting the proper frame from SMD simulation, the relaxation of GS-APP structure in MD simulation in Continue mode can bring the scissile bond (carbonyl group of V30 residue) to the catalytic residues. As seen in Figure 7C, the violin plots indicate that short distances between the scissile bond and one of catalytic residue (D257) are reachable in all MD simulations. The most frequent distance is in range 4–5 Å indicating that such interaction can be bridged by water molecule although no explicit water molecules are present in the simulations. Bridging by water molecules was found by Bhattarai *et al.* (11) in their GaMD (Gaussian-accelerated MD) simulations of GS-APP in explicit environments. Since the water molecule is required for the cleavage our results are in agreement with those from much more sophisticated approaches.

**Figure 8.**
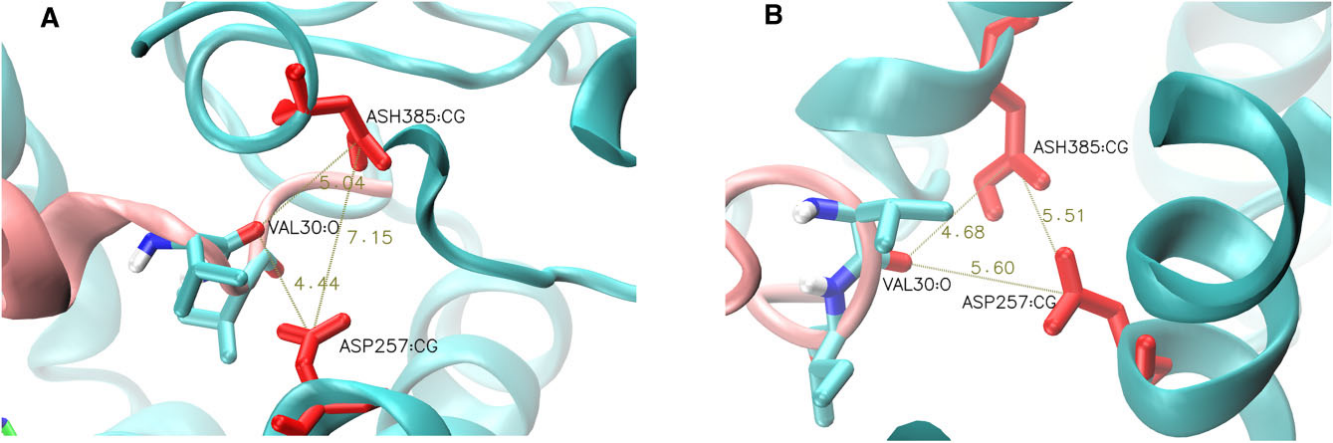
Conformations of APP-GS with shortest distance of the second cleavage site (Val30) to the catalytic residues. (**A**) for WT. (**B**) for PS-1 K380E mutant.

Recently, Suzuki *et al.* (15) investigated specific mutations near the APP cleavage site that influence Aβ production. Most of those mutations were located after the first (epsilon) cleavage site and therefore were not suitable for GS-SMD. Therefore, we decided to study APP mutation T714I (T27 using GS-SMD numbering) and PS-1 mutation K380E and compare to WT. From each SMD trajectory, we chose a frame where a distance of the next cleavage site to the catalytic residues was the shortest. We considered trimming of Aβ_49_ peptide to Aβ_46_, that requires a cleavage at V717 (V30 in GS-SMD). We compared the SMD work required to pull the substrate into a conformation that facilitate cleavage (Figure 8). In all cases it was possible to bring V30 to the catalytic residues but the required work was larger for APP T714I: 50 (±20) kJ/mol for WT, and 94 (±52) kJ/mol for the mutant. The difference in the mean values of work is statistically significant. Our results are consistent with experimental data showing that this mutation considerably reduces the cleavage activity of GS. For PS-1 mutation K380E the mean work was 98 (±81) kJ/mol. A high standard deviation may suggest that the mutation causes certain destabilization of the active site—it is also not statistically different from mean work for WT. However, this is also in agreement with experiment since PS-1 K380E mutant does not change GS activity unless associated with APP mutations. Details of simulations are shown in Supplementary material. The SMD works required to pull the substrate into a conformation that facilitate cleavage are shown in Supplementary Table S1, while the force and work charts for particular SMD simulations are shown in Supplementary Figures S1–S6.

In conclusion, the GS-SMD server allows to quickly obtain the results of unfolding of any substrate of GS in the active site of this protease. The system under study is built by selecting the required building blocks and there is no need to add membrane, water and equilibrate the system. The server can be used to explore mechanisms of trimming and unfolding the substrate in order to obtain quick but reliable results for later verification in MD/SMD simulations in the explicit environments or with experimental methods. The GS-SMD server can also be used as a database of currently available GS structures and as a useful database of GS substrates divided into subgroups based on their TM sequences.

## DATA AVAILABILITY

GS-SMD is free for all users without logging in. E-mail is optional (for receiving notification of job completion) and is not stored on server after sending notifications. All conducted simulations described in this paper are available on GS-SMD server as Shared Jobs or Example Jobs.

## Supplementary Material

gkad409_Supplemental_File
