# Supplementary material for: GS-SMD server for steered molecular dynamics of peptide substrates in the active site of the γ-secretase complex
Source: Nucleic Acids Res. 2023 May 19;51(W1):W251–62. doi: 10.1093/nar/gkad409 (PMC10320170; doi:10.1093/nar/gkad409)
Supplement: gkad409_Supplemental_File [file gkad409_supplemental_file.pdf]

Supplementary material

GS-SMD server for steered molecular dynamics of peptide substrates in the active site of the γ-secretase complex

Urszula Orzeł[1#], Paweł Pasznik[1#], Przemysław Miszta[1], Marcin Lorkowski[1], Szymon Niewieczerzał[1], Jakub Jakowiecki[1], Sławomir Filipek[1*]

[1] Faculty of Chemistry, Biological and Chemical Research Centre, University of Warsaw, Warsaw, Poland.

[#] The authors wish it to be known that, in their opinion, the first two authors should be regarded as joint First Authors

[*] To whom correspondence should be addressed.

SUPPLEMENTARY DISCUSSION

We performed three series of SMD simulations in GS-SMD server, 8 for each case: WT, APP mutation T714I and PS-1 mutation K380E. We used default settings: simulation in "all" mode (whole structure of GS), pulling APP at residue no. 33 (Leu720), simulation 15ns long, spring constant k = 1.0 kcal/mol/Å^2, and pulling velocity v = 0.1 m/s. From each trajectory, we chose a frame where a distance of the next cleavage site to the catalytic residues was the shortest, and short enough for the cleavage to happen: a sum of distances between backbone Val30:O atom to sidechain CG atoms of catalytic residues Asp257 and Asp385 were below 12.5 Å; and a distance between sidechain CG atoms of catalytic residues was below 8 Å (see Figure 8 in main text). We considered trimming of Aβ_{49} peptide to Aβ_{46}, that requires a cleavage at Val717 (Val30 using GS-SMD numbering). We compared the SMD work required to pull the substrate into a conformation that facilitate cleavage. The results are summarized in Table S1. The force and work charts for particular SMD simulations are shown in Figures S1-S6.

For WT the mean work required to pull the substrate to the next cleavage conformation is 50.4 ± 20.3 kJ/mol, while for APP mutation T714I mean work is 93.5 ± 51.9 kJ/mol. The difference of means is equal to 43.1 kJ/mol and is statistically significant ($p = 0.0458 < 0.05$).

This result suggests that for the APP substrate with mutation T714I the energy barrier to unwind the substrate and provide the residue of the second cleavage site close to the catalytic residues of GS is higher. Our results are consistent with experimental data (1) showing that this mutation significantly reduces the cleavage activity of GS.

We also analyzed the impact of PS-1 mutation K380E on a system. The mean work in case of mutation K380E is 98.4 ± 80.9 kJ/mol. High standard deviation represents high dispersion and may suggest that the mutation causes certain destabilization of the active site. The difference between mean work in WT system and mutation K380E equals 48 kJ/mol but does not meet a criterion to have statistical significance, $p = 0.1256 > 0.05$. This is also in agreement with experiment since PS-1 K380E mutant does not change GS activity unless associated with APP mutations.

REFERENCES

1. Suzuki, R., Takahashi, H., Yoshida, C., Hidaka, M., Ogawa, T. and Futai, E. (2023) Specific Mutations near the Amyloid Precursor Protein Cleavage Site Increase gamma-Secretase Sensitivity and Modulate Amyloid-beta Production. *Int. J. Mol. Sci.*, **24**, 3970.

https://doi.org/10.3390/ijms24043970

https://www.ncbi.nlm.nih.gov/pmc/articles/PMC9959964

https://pubmed.ncbi.nlm.nih.gov/36835396

Table S1. The SMD works required to pull the substrate into a conformation that facilitate cleavage (sum of distances from a cleavage site to the catalytic residues is the shortest). Wild Type (APP WT), APP mutation T714I and GS PS-1 mutation K380E.

Wild Type (WT)

repetition	Frame	Time [ns]	Work [kJ/mol]	Val30-Asp257 [Å]	Val30-Asp385 [Å]	Sum of distances [Å]	Asp257-Asp385 [Å]
**1**	32	9.9	**79.2**	4.44	5.04	9.48	7.15
**2**	28	8.7	**40.8**	4.33	5.83	10.16	7.57
**3**	31	9.6	**76.6**	4.4	4.98	9.38	6.87
**4**	26	8.1	**28.9**	3.83	5.5	9.33	5.98
**5**	31	9.6	**60.2**	6.44	4.89	11.33	7.23
**6**	27	8.4	**35.8**	6.3	3.51	9.81	6.9
**7**	18	5.7	**28.4**	4.41	3.76	8.17	6.16
**8**	25	7.8	**53.2**	5.83	4.74	10.57	6.03
		**Mean:**	**50.4**				
		**STDEV:**	**20.3**				

APP mutation T714I

repetition	Frame	Time [ns]	Work [kJ/mol]	Val30-Asp257 [Å]	Val30-Asp385 [Å]	Sum of distances [Å]	Asp257-Asp385 [Å]
**1**	32	9.9	**98.9**	7.42	5.05	12.47	7.48
**2**	27	8.4	**54.5**	5.04	4.06	9.1	7.96
**3**	34	10.5	**61.2**	5.93	4.11	10.04	7.13
**4**	30	9.3	**107.1**	4.47	6.73	11.2	7.73
**5**	34	10.5	**83.4**	6.21	5.31	11.52	6.64
**6**	42	12.9	**213**	5.03	4.42	9.45	6.18
**7**	32	9.9	**69.7**	4.46	4.43	8.89	6.03
**8**	33	10.2	**60.4**	3.98	4.82	8.8	7.19
		**Mean:**	**93.5**				
		**STDEV:**	**51.9**				

GS PS-1 mutation K380E

repetition	Frame	Time [ns]	Work [kJ/mol]	Val30-Asp257 [Å]	Val30-Asp385 [Å]	Sum of distances [Å]	Asp257-Asp385 [Å]
**1**	14	4.5	**23.9**	5.6	4.68	10.28	5.51
**2**	40	12.3	**87.4**	5.84	4.84	10.68	6.62
**3**	39	12	**193.2**	5.62	5.18	10.8	7.23
**4**	35	10.8	**73.2**	4.72	6.93	11.65	7.36
**5**	28	8.7	**63.8**	5.8	4.63	10.43	6.85
**6**	20	6.3	**40.5**	4.02	5.74	9.76	7.63
**7**	34	10.5	**52.5**	5.89	6.08	11.97	5.53
**8**	48	14.7	**253**	5.81	4.96	10.77	5.26
		**Mean:**	**98.4**				
		**STDEV:**	**80.9**				

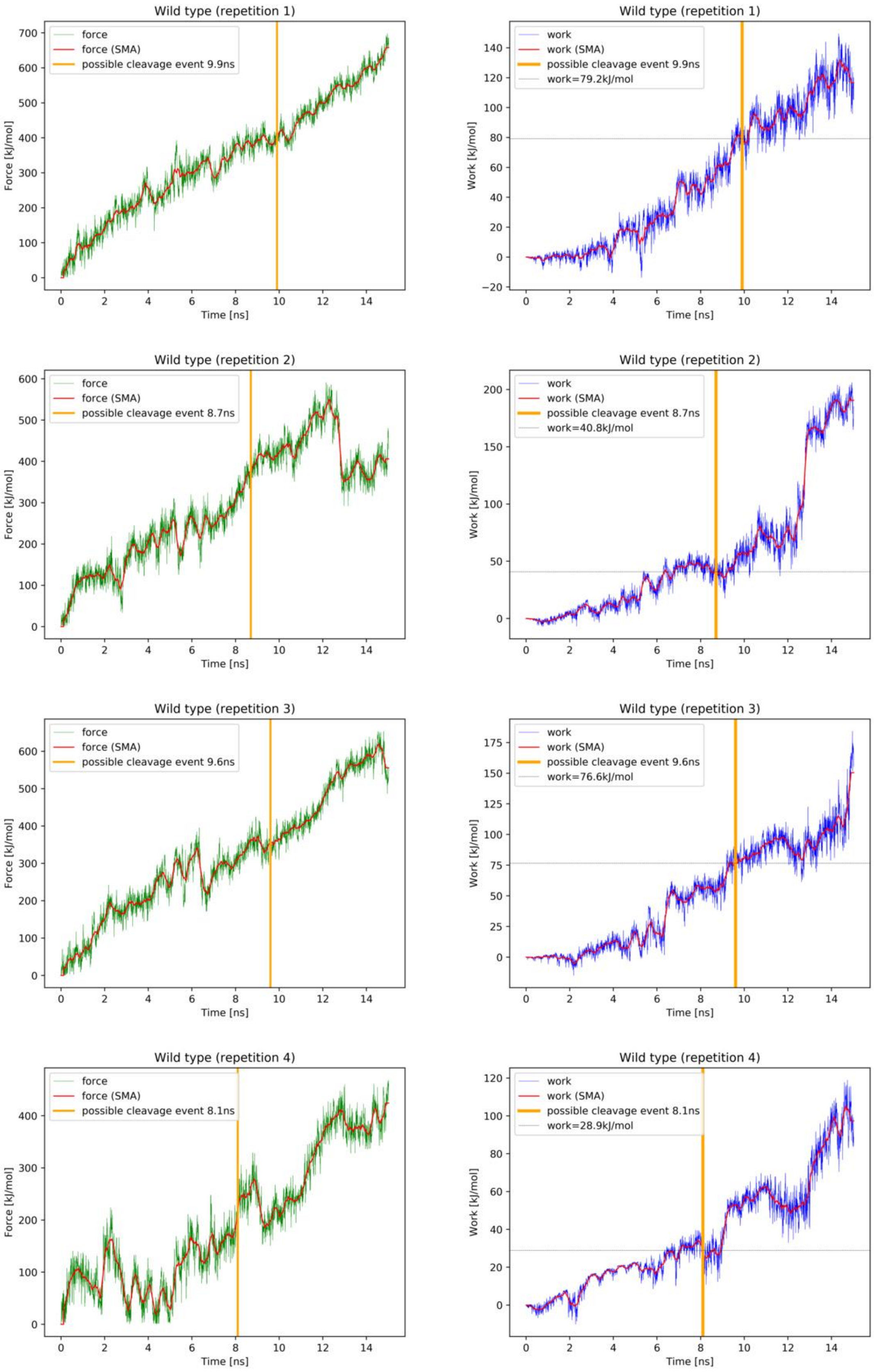

Figure S1. Force and work in SMD simulation of GS-APP WT complex. A time frame with the shortest distance between catalytic residues and substrate APP Val30 was shown with an orange line, indicating a possible cleavage event. Repetitions 1–4.

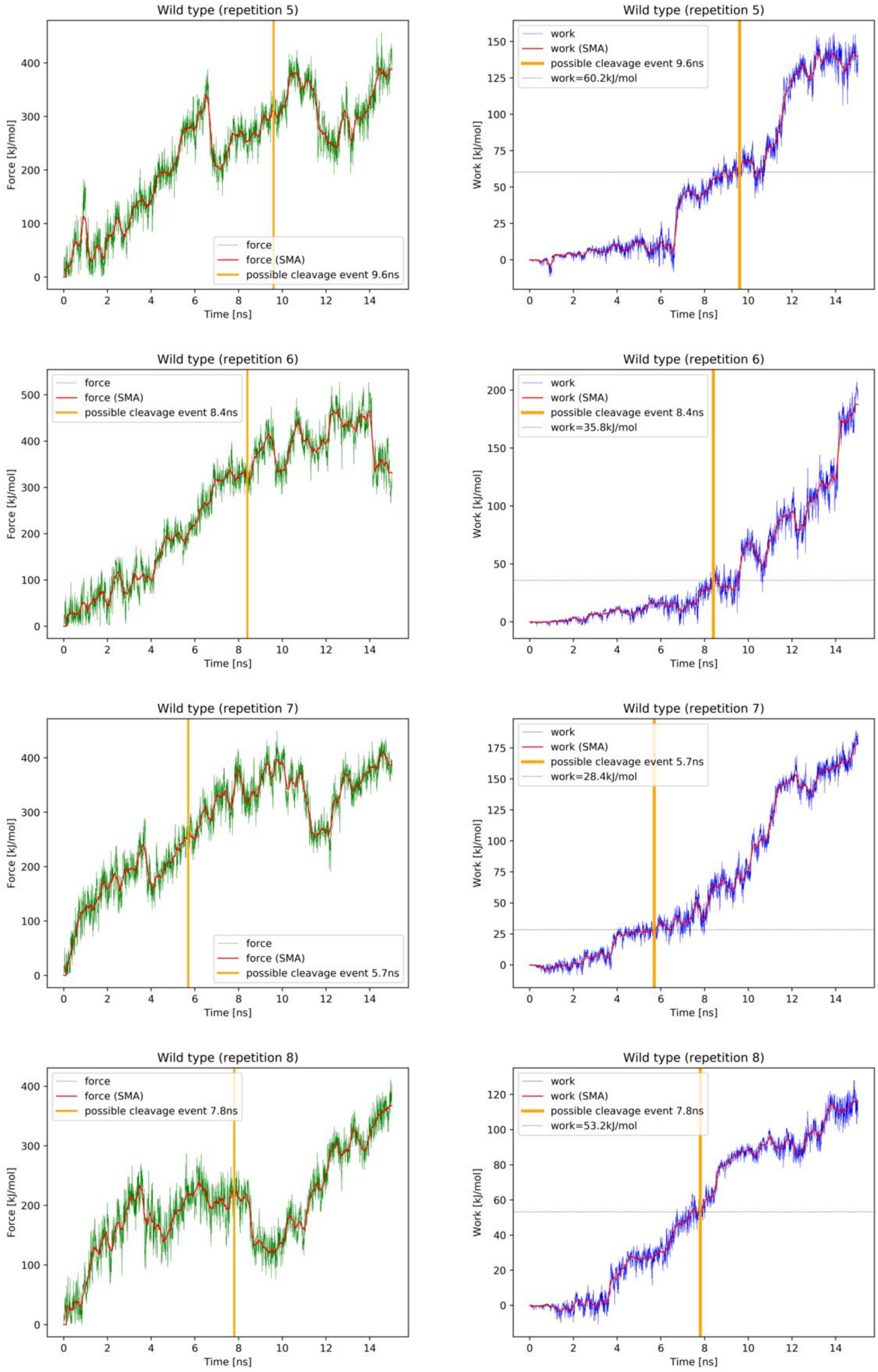

Figure S2. Force and work in SMD simulation of GS-APP WT complex. A time frame with the shortest distance between catalytic residues and substrate APP Val30 was shown with an orange line, indicating a possible cleavage event. Repetitions 5–8.

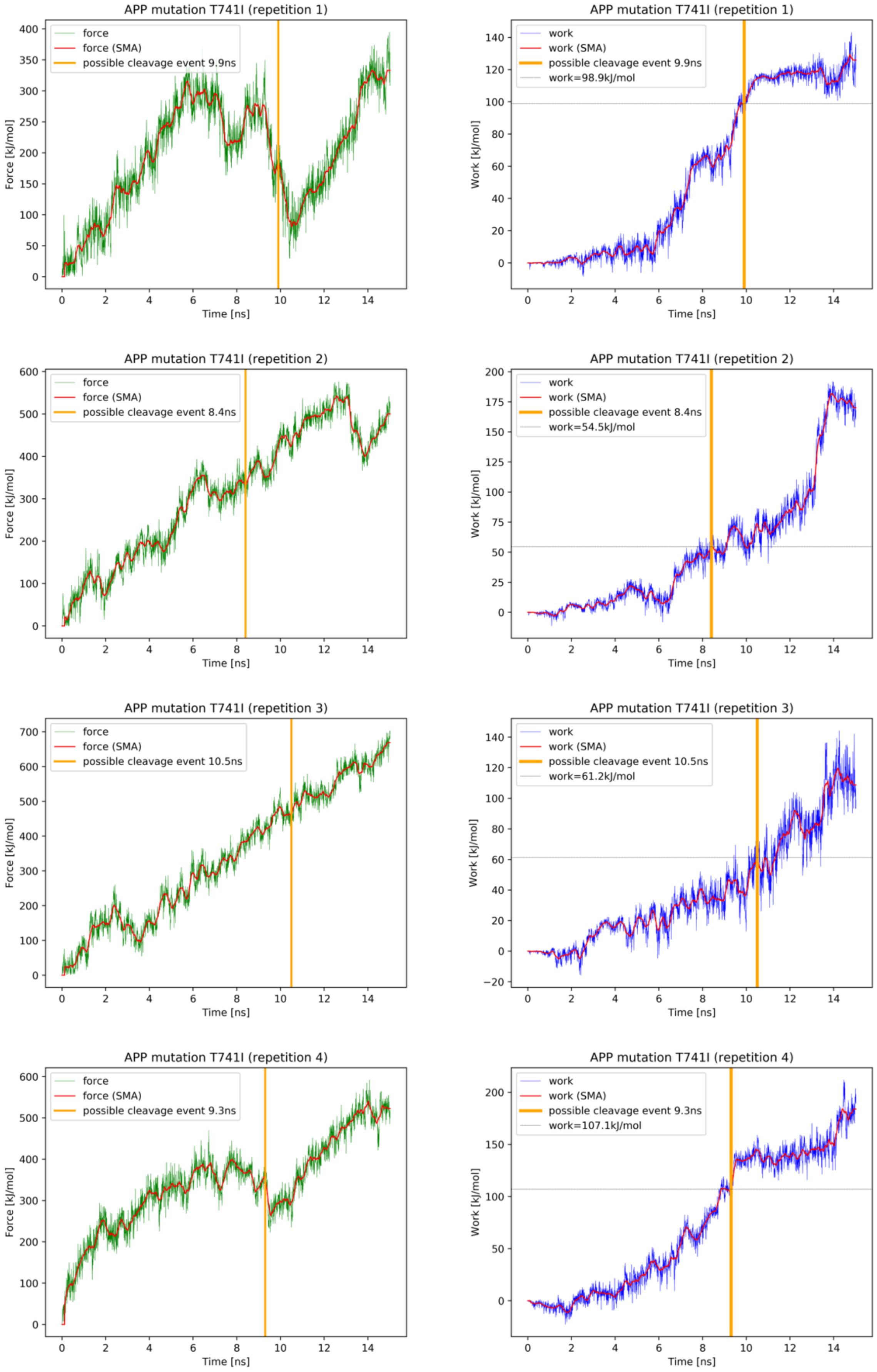

Figure S3. Force and work in SMD simulation of GS-APP complex with APP mutation T714I. A time frame with the shortest distance between catalytic residues and substrate APP Val30 was shown with an orange line, indicating a possible cleavage event. Repetitions 1–4.

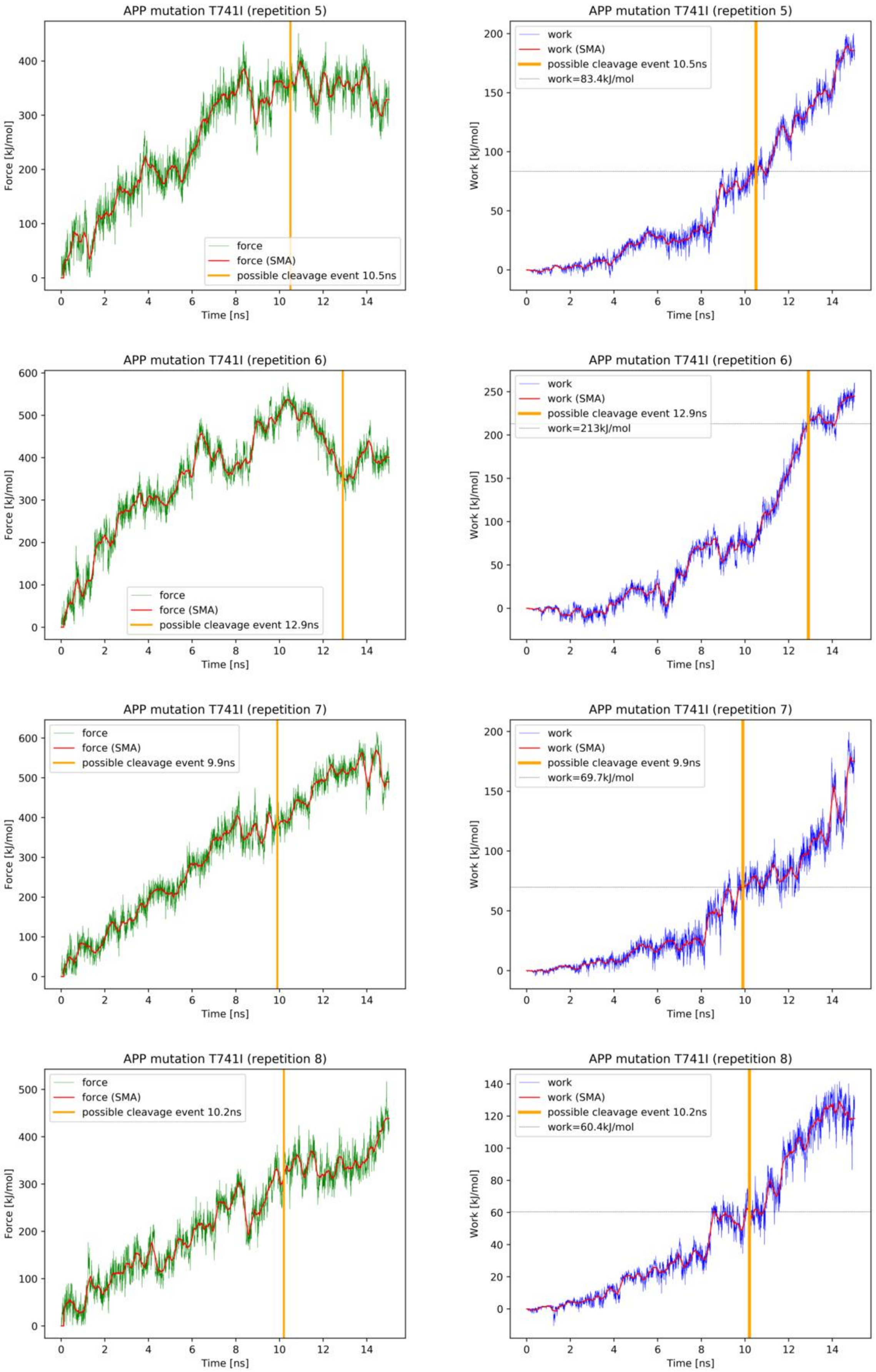

Figure S4. Force and work in SMD simulation of GS-APP complex with APP mutation T714I. A time frame with the shortest distance between catalytic residues and substrate APP Val30 was shown with an orange line, indicating a possible cleavage event. Repetitions 5–8.

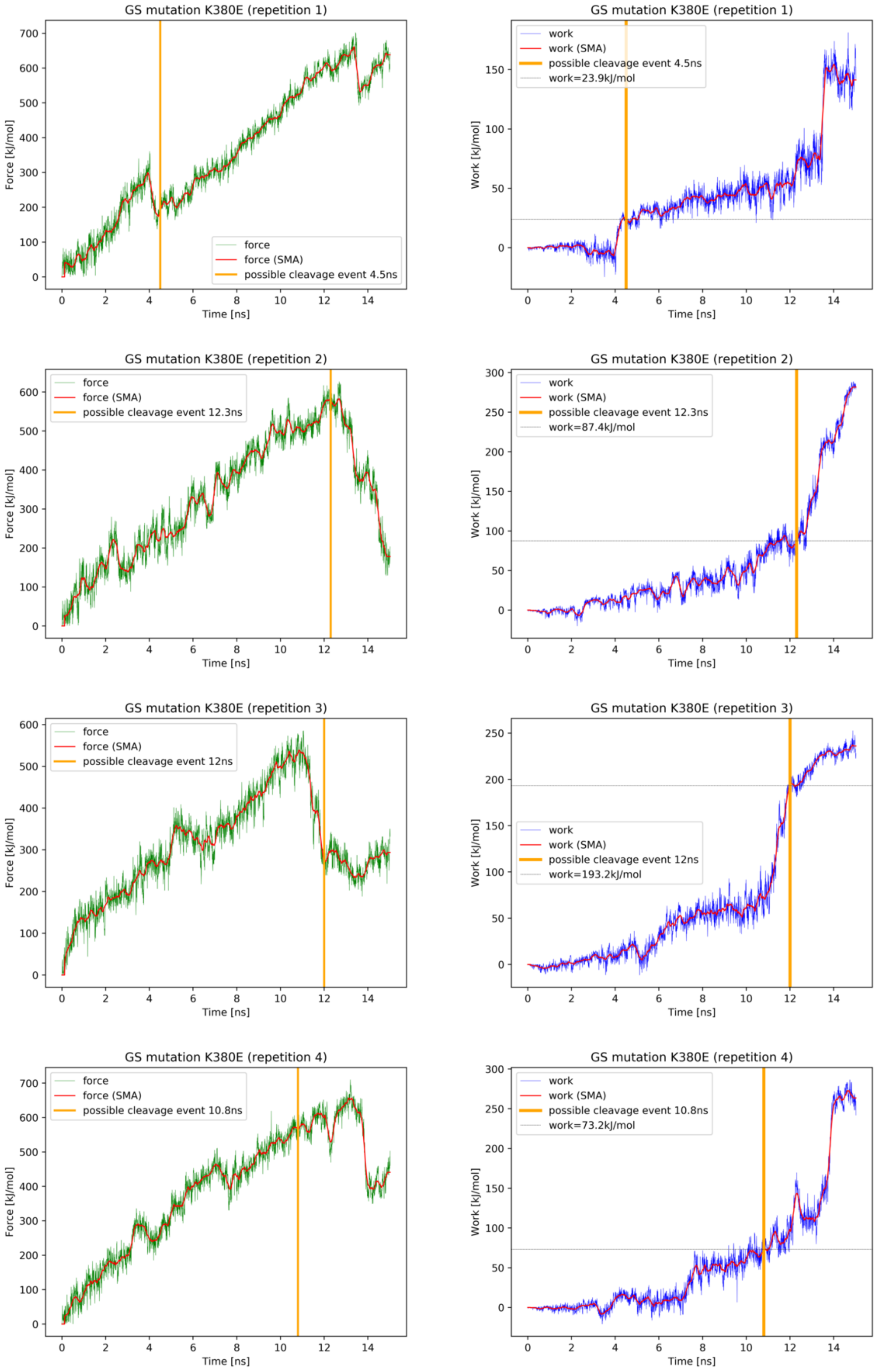

Figure S5. Force and work in SMD simulation of GS-APP complex with GS PS-1 mutation K380E. A time frame with the shortest distance between catalytic residues and substrate APP Val30 was shown with an orange line, indicating a possible cleavage event. Repetitions 1–4.

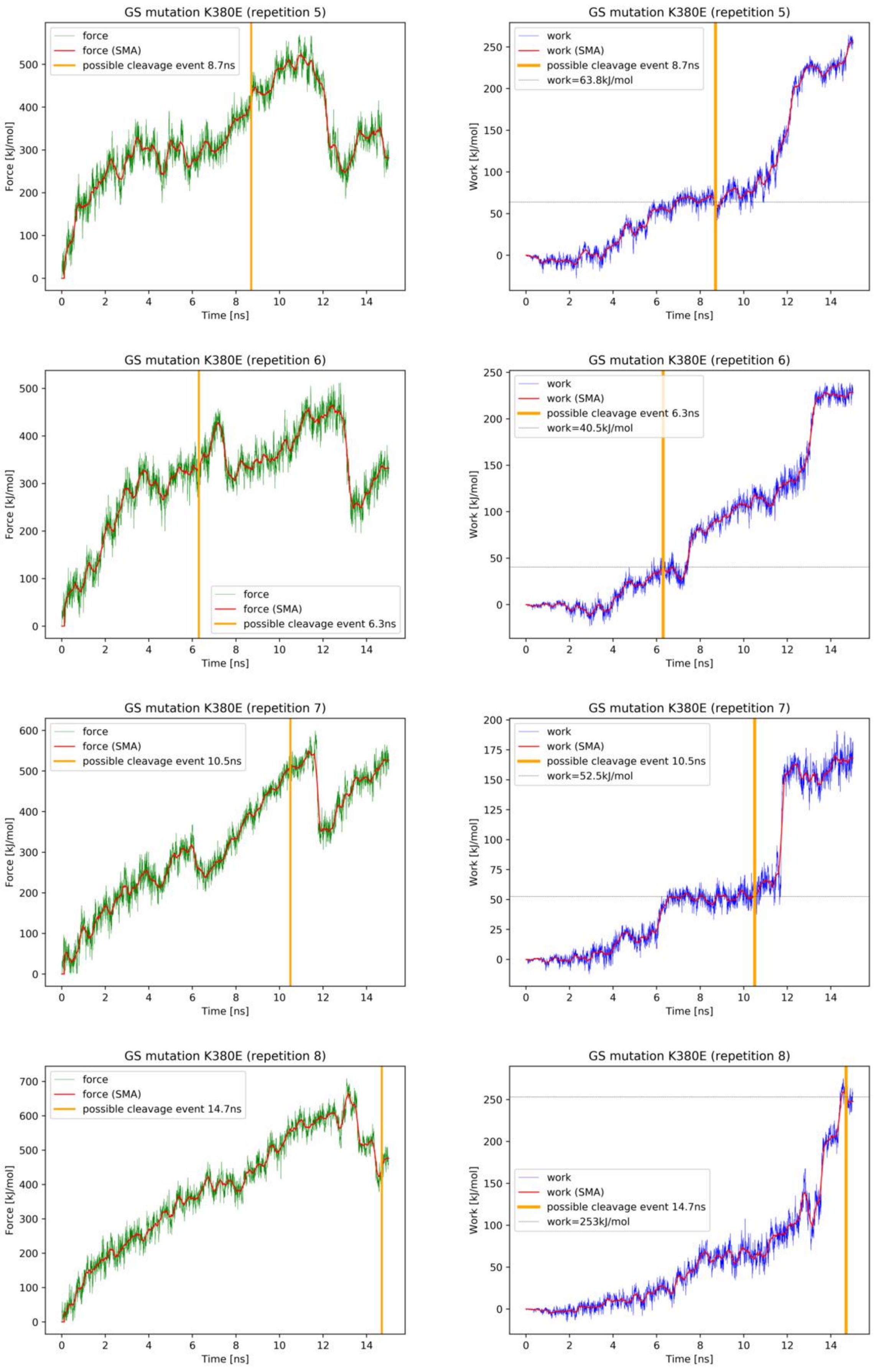

Figure S6. Force and work in SMD simulation of GS-APP complex with GS PS-1 mutation K380E. A time frame with the shortest distance between catalytic residues and substrate APP Val30 was shown with an orange line, indicating a possible cleavage event. Repetitions 5–8.
